# Antiparasitic effect of Farnesol against *Leishmania major*: A rationale from *in vitro* and *in silico* investigations

**DOI:** 10.1371/journal.pone.0293290

**Published:** 2023-11-06

**Authors:** Harshita Sharma, Rakesh Sehgal, Nishant Shekhar, Gaurav Shoeran, Upninder Kaur, Bikash Medhi

**Affiliations:** 1 Department of Medical Parasitology, PGIMER, Chandigarh, India; 2 Aarupadai Veedu Medical College & Hospital, Vinayaka Mission’s Research Foundation-DU, Puducherry, India; 3 Department of Medical Microbiology, PGIMER, Chandigarh, India; 4 Department of Pharmacology, PGIMER, Chandigarh, India; Iran University of Medical Sciences, ISLAMIC REPUBLIC OF IRAN

## Abstract

Leishmaniasis is a vector-borne parasitic infection caused by the infective bite of female Phlebotomine sandflies. Treatment of leishmaniasis by conventional synthetic compounds is met by challenges pertaining to adverse effects which call for the discovery of newer anti-leishmanial molecules. This study was performed to evaluate the effect and modes of action of a sesquiterpene alcoholic molecule Farnesol on Leishmania major, the causative agent of Zoonotic CL. The cytotoxic effect of Farnesol against *L*.*major* promastigotes, amastigotes and macrophages was assessed by MTT test and counting. The IC_50_ on promastigotes by Farnesol on *L*.*major* was also evaluated by flow cytometry. In the findings, promastigotes were reduced at 167μM. The mean numbers of *L*.*major* amastigotes in macrophages were significantly decreased on exposure to Farnesol at 172μM. In addition, Farnesol induced significant apoptosis dose-dependent on *L*.*major* promastigotes. *In silico* protein-ligand_binding analyses indicated the effect of Farnesol in perturbation of the ergosterol synthesis pathway of *Leishmania* with attributes suggesting inhibition of Lanosterol-α-demethylase, the terminal enzyme of ergosterol synthesis machinery. Findings from flow cytometry reveal the role of Farnesol in apoptosis-induced killing in promastigotes. Farnesol was effective at very lower concentrations when compared to Paromomycin. Further studies are crucial to evaluate the therapeutic potential of Farnesol alone or in combination with other conventional drugs in animal models.

## 1 Introduction

Leishmaniasis is a vector-borne parasitic infection caused by the infective bite of female Phlebotomine sandflies [1]. World Health Organization (WHO) estimates 100,000 cases to be reported annually on a global scale, moreover, In 2021, leishmaniasis was reported in 99 countries, including those endemics for both VL and CL (n = 71), VL only (n = 9), and CL only (n = 19). By February 2023, 66% of VL-endemic and 61% of CL-endemic countries provided data. Eight countries contributed to 89% of VL cases, while nine countries contributed to 88% of CL cases. The global count for imported cases in 2021 included 385 CL and 60 VL cases [2]. However, the annual estimated incidence of CL alone ranges from 0.7 to 1 million cases, indicating a significant threat posed by CL [3]. There are six clinical types of leishmaniasis: CL, mucocutaneous leishmaniasis (MCL), diffuse cutaneous leishmaniasis (DCL), visceral leishmaniasis (VL), post-kala-azar dermal leishmaniasis (PKDL), and leishmaniasis recidivans (LR). The symptoms might range from mild ulceration with self-healing skin sores to immune complications leading to death [4]. CL, caused by various *Leishmania* species such *as L*.*aethiopica*, *L*.*tropica*, *and L*.*major*, presents as self-healing ulcers known as "oriental sore". The lesions can be localized or disseminated, and typically resolve spontaneously within a few months in individuals with intact immune systems.

*L*.*major*, which belongs to the *L*.*tropica* complex, is responsible for Old World CL. It predominantly affects rural areas, earning the name "rural zoonotic CL". The disease is primarily reported in the Middle East, India, China, Central Africa, Central and South America, and Central and Western Asia [5]. In India, CL outbreaks have mainly been documented in arid regions such as Rajasthan, Bikaner, and Gujarat, with sporadic cases reported from Punjab, Assam, and Haryana. However, recent reports have indicated CL cases in other parts of the country, including Himachal Pradesh and Kerala [6–8].

The current CL treatment strategies include the administration of antimony-based drugs, Glucantime, Paromomycin, and Miltefosine [9]. However, these compounds are associated with a number of adverse effects that limits their usage, like systemic side effects, toxicities, drug resistance, and painful injections which leads to a reduction in patient acceptance [10]. Besides being expensive these are also long and tiring therapies. Patients can suffer damage to their hearts, livers, pancreas, hematopoietic tissues, and renal systems when these compounds fail to provide coverage against *Leishmania*. As a result, it is critical to introduce compounds with fewer complications for CL patients [11,12]. Compounds with natural antibacterial, anticancer, anti-inflammatory properties, including anti-leishmanial properties, contribute to the popularity of alternative methods [13,14].

Farnesol is one such natural compound derived from a range of plants such as citronella, cyclamen, balsam, musk while it is also a constituent of many essential oils [15–18]. Farnesol shows anti-cancer effects on several forms of cancers such as prostate cancer and lung cancer etc. In addition, to being identified as a quorum-sensing molecule of *Candida albicans*, it induces cell death above physiological concentrations which were also observed against bacterial species such as *Staphylococcus aureus*, *Streptococcus mutans* and the plant pathogenic fungus *Fusarium graminearum* [19]. Farnesol also regulates the anti-inflammatory responses which have been reported in asthma, edema, gliosis and skin tumors [20]. It has been reported to exhibit significant antimicrobial properties against *Plasmodium* causing malaria and *Toxoplasma* [21]. Now, given these background evidences of the anti-inflammatory, anti-cancerous, antimicrobial and antiprotozoal effects of Farnesol it might also be possible that the drug shows inhibitory effects against *Leishmania spp*. Hence, we designed the current study to screen the activity and find a probable target for action of Farnesol against an Indian standard strain of *Leishmania major* (MHOM/SU/73/5ASKH)*in silico* and *in vitro*.

## 2 Materials and methods

### 2.1 Chemical preparation

Farnesol (Catalog No. F203) and Paromomycin were purchased from Sigma-Aldrich Co. Germany (Catalog No. P-5057). To achieve desired concentrations (100–560 μM), Farnesol and Paromomycin were dissolved in absolute alcohol and sterile distilled water, respectively and then further dissolved in RPMI 1640 media for combination studies. For the assessment of any combinatory effect of Farnesol and Paromomycin, concentrations of 100+100, 165+350, 180+390, and 300+300μM were also prepared.

### 2.2 Promastigotes culture & promastigotes assay

*L*.*major*promastigotes (strain MHOM/SU/73/5ASKH) were grown in RPMI 1640 supplemented with 10% heat-inactivated Fetal Bovine Serum (Gibco, New York, NY, USA) at 28°C, 500 μl Pen-Strep containing 100 IU/ml penicillin and 100 μg/ml streptomycin was used **[22]**. Logarithmic growth-phase promastigotes of *L*.*major* (1× 10^6^ ml^−1^ cells) were cultured for 72 hours at 28°C in 96-well plates (Thermofisher Scientific) in the presence of various concentrations of Farnesol & Paromomycin as control drug (110–560μM). Promastigotes were counted directly in the Neubauer chamber under a light microscope after 24, 48, and 72 hours of incubation for counting assay and for MTT Assay after drug treatment for 72 hours at 28°C, 20 μl MTT [3-(4, 5-dimethylthiazol-2-yl)-2,5-diphenyltetrazolium bromide] at 5 mg ml^−1^ concentration was added to each well and incubated for another 4 hours (Table 1). Centrifugation was used to remove the medium, and 100 μl dimethyl sulfoxide (DMSO) was added to each well. The MTT was measured using an ELISA reader at 540 nm. The study comprised both negative and positive controls using Paromomycin at 350 μM. All experiments carried out as triplicate **[23]**.

**Table 1:** The table shows mean promastigote viability after drug treatment at various drug concentrations (‘-‘No live promastigotes observed under light microscope).

Concentrations of Farnesol (μM)	(Mean±SD)
110	310±38.4
130	233±52.5
150	211±20.3
170	179±13.2
190	142.6±30.22
210	86.6±22.03
230	90.3±14.64
250	46±8.88
260	33.66±4.93
270	19.3±7.63
360	-
460	-
560	-
Positive Control	375.6±1.52
Paromomycin @ at 332 μM	286±7

### 2.3 Flow cytometry analysis

The current work employed flow cytometry to determine the likely effective concentration of Farnesol & Paromomycin as a control (100–400μM) on promastigotes using double labelling with annexin V-FLUOS and propidium iodide (PI). In brief,1×10^6^ ml^−1^ promastigotes treated with Farnesol and those untreated were rinsed twice with cold PBS solution and centrifuged for 10 minutes at 1500 g. The promastigotes were then incubated for 15–20 minutes at 25°C in the dark with 5 μl of annexin-V FLUOS in the presence of 5 μl PI plus 500 μl buffer. Finally, the samples were examined using the FACS Calibur flow cytometer (FACS Canto II). The data was analysed using Flow Jo software, and the percentages of necrotic, apoptotic, and normal cells were calculated **[24]**.

### 2.4 Macrophage culture

THP-1 Cell line was obtained from Cell Repository, National Centre for Cell Culture (NCCS) Pune, India & cultured in RPMI 1640 supplemented with 10% Fetal Bovine Serum and 100 IU ml^−1^ penicillin and 100 μg ml^−1^ streptomycin and incubated in a CO_2_ incubator (37°C, 5% CO_2_, and 95% relative humidity).

### 2.5 Cytotoxicity assay

The monocytic cells were cultured in 6 well plates for 72 h with RPMI 1640 and PMA treated for differentiation and placed in a CO_2_ incubator (37°C, 5% CO2, and 95% relative humidity). The cells were then trypsinized and seeded in 96 well plates containing Farnesol at concentrations of 100–1000μM. After 72 h of drug action, 100 μl of 5 mg/ml MTT dye was added. After a 4h incubation with MTT dye and breakdown of formazan crystals with DMSO, readings at 570 nm were obtained. For MTT test readings, a Tecan i-control, 2.0.10.0 reader was used [22].

### 2.6 Amastigotes assay

**Fig 1:**
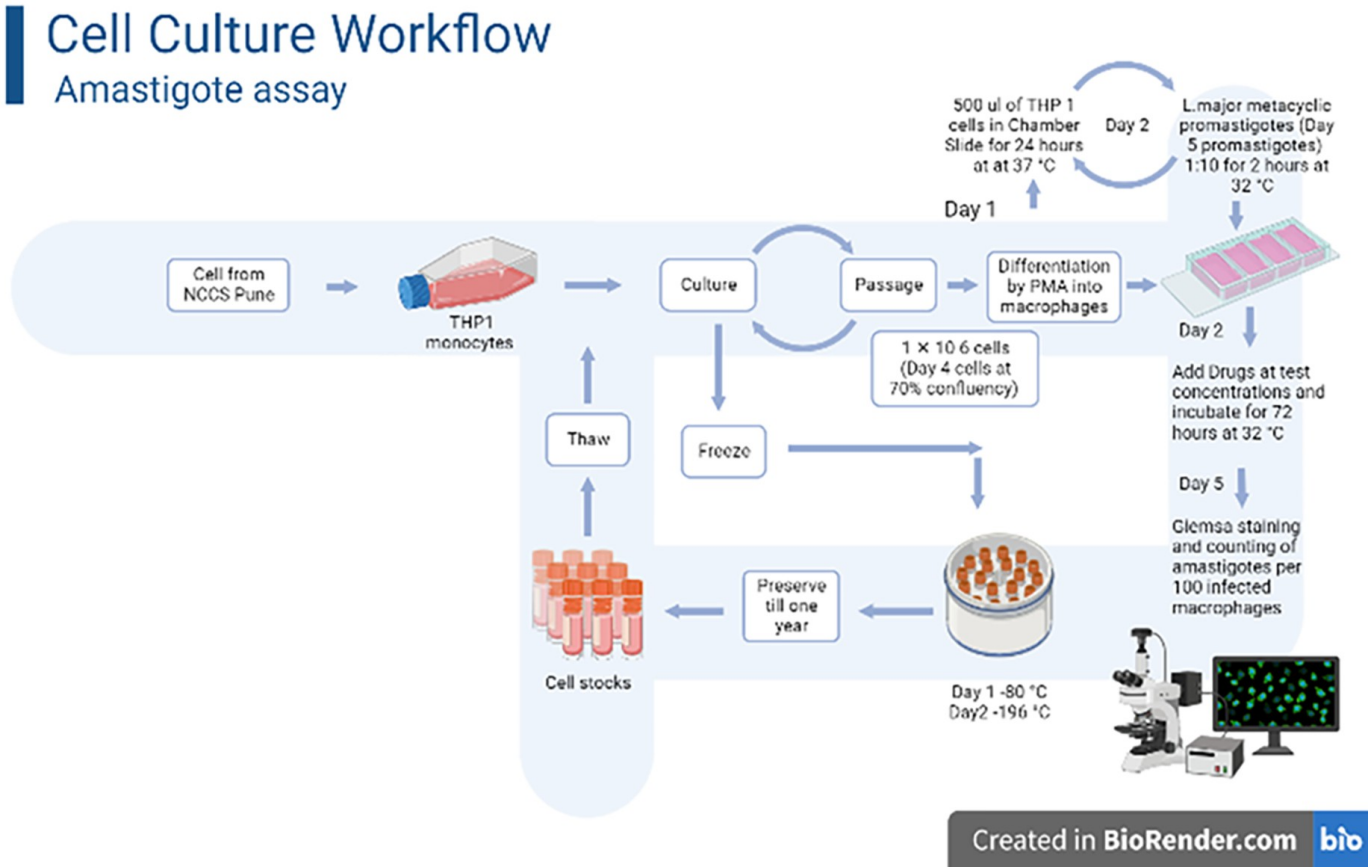
Cell culture and amastigote assay workflow- THP 1 monocyte were cultured and passaged every alternate day for drug testing experiments: Day 1–500 μl of THP 1 PMA treated cells were inoculated in chamber slide for 24 hours at 37°C for differentiation in macrophages; Day 2- *Lmajor* metacyclic promastigotes were infected into these macrophages and incubated for phagocytoses at a ratio of 1:10 at 32°C for 2 hours. Fresh media was poured to phagocytized macrophages with differing drug concentrations for 72 hrs at 32°C. Day 5- Giemsa staining and counting of amastigotes per 100 infected macrophages is performed under light microscope. Cryopreserved cells at 196°C can be revived for a period of 1 year.

The macrophage cells were initially grown in 4-well chamber slides with RPMI 1640 for 24 hours before being put in a CO_2_ incubator (37°C, 5% CO2, and 95% relative humidity). After that, the medium was removed from the culture and stationary phase promastigotes were inoculated at a 1:10 macrophage to promastigote ratio. The macrophages and promastigotes were cultured for another 2 hours at 32°C to allow for phagocytosis. After 2 hours, the excess parasites were washed away. The cells were then cultivated in new media with Farnesol at different concentrations (100–560μM), and incubated at 32°C. Paromomycin (100–560μM), was used as a positive control. After 72 hours of drug treatment, the slides were methanol-fixed and stained with Giemsa (Fig 1). Light microscopy was used to calculate the number of amastigotes per 100 macrophages [24].

### 2.7 Combination therapy

Different concentrations of Farnesol and Paromomycin (100+100, 165+350, and 300+300 μM) were combined and cultured with *L*.*major* log phase promastigotes for 24 hours at 28°C for anti-promastigote & amastigote assays.

### 2.8 *In silico* drug-binding investigation

Upon relevant literature search, the binding affinity of Farnesol was tested against the key enzymes of the ergosterol synthesis pathway i.e., Farnesol pyrophosphate synthase (FPPS) [PDBid: 4K10], Squalene synthase [modbase model], and Lanosterol 14-demethylase (CYP51) [modbase model]. The binding of arnesol was assessed based on outcomes generated from molecular docking, MM-GBSA-based ΔG_*bind*_ calculation and molecular dynamics (MD) analysis. All *in silico* operations were performed on Schrödinger Maestro and associated modules i.e., Glide for docking, Prime for MM-GBSA and Desmond for MD simulations and trajectory analysis.

### 2.9 Statistical Analysis

One-way ANOVA were used to analyze mean values. The experimental data were summarized using mean ± SEM. The statistically significant level for differences between mean values was accepted at P < 0.05. Graph pad 9.3.1 was employed for statistical analyses.

## 3. Results

### 3.1 Promastigote assay

**Fig 2:**
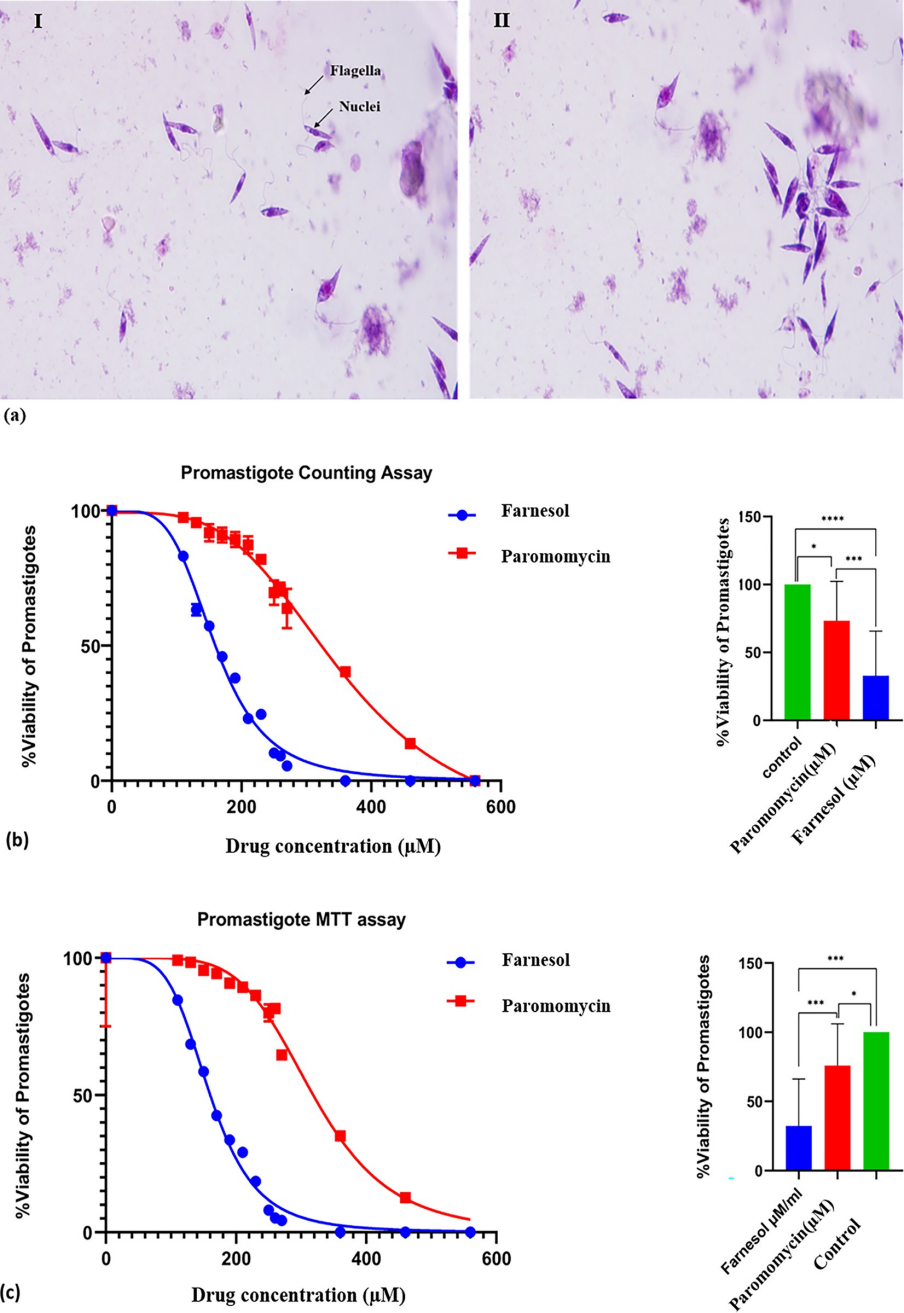
**a.** Promastigotes by Giemsa staining under light microscopy **b.** Graph with IC 50 curve by Promastigote counting assay **c.** IC 50 graph by Promastigote MTT assay. All statistical tests were two-tailed, with a significance level of p < 0.05,* p<0.05,** p<0.01, *** p <0.001 & **** p<0.0001 was considered statistically significant.

The anti-leishmanial action of Farnesol (110–550μM) was observed using light microscopy by assessing the number of parasites present upon treatment (Table 1). Farnesol’s inhibitory effects on promastigotes are dosage and time dependent, which means that percentage killing increases depending on period of exposure and concentrations of Farnesol demonstrating anti-promastigote effects of Farnesol with an IC_50_ value of 167.6 ± 4.5μM/ml and IC_90_ at 273.10 ± 2.44 μM by MTT assay and by counting assay- IC_50_ at 170.5 ± 4.2μ*M* and IC_90_ at 287.69 ± 2.46 μ*M* whereas for Paromomycin (110–550μM)—IC_50_ at 332.0 ± 5.1μ*M* & IC_90_ at 510.79 ± 2.71 μ*M* for MTT & IC_50_ at 353.2 ± 3.7μM & IC_90_ at 639.62 ± 2.81μM for counting assay respectively (Table 1) (Fig 2).

### 2.2 Flow cytometry assay

**Fig 3:**
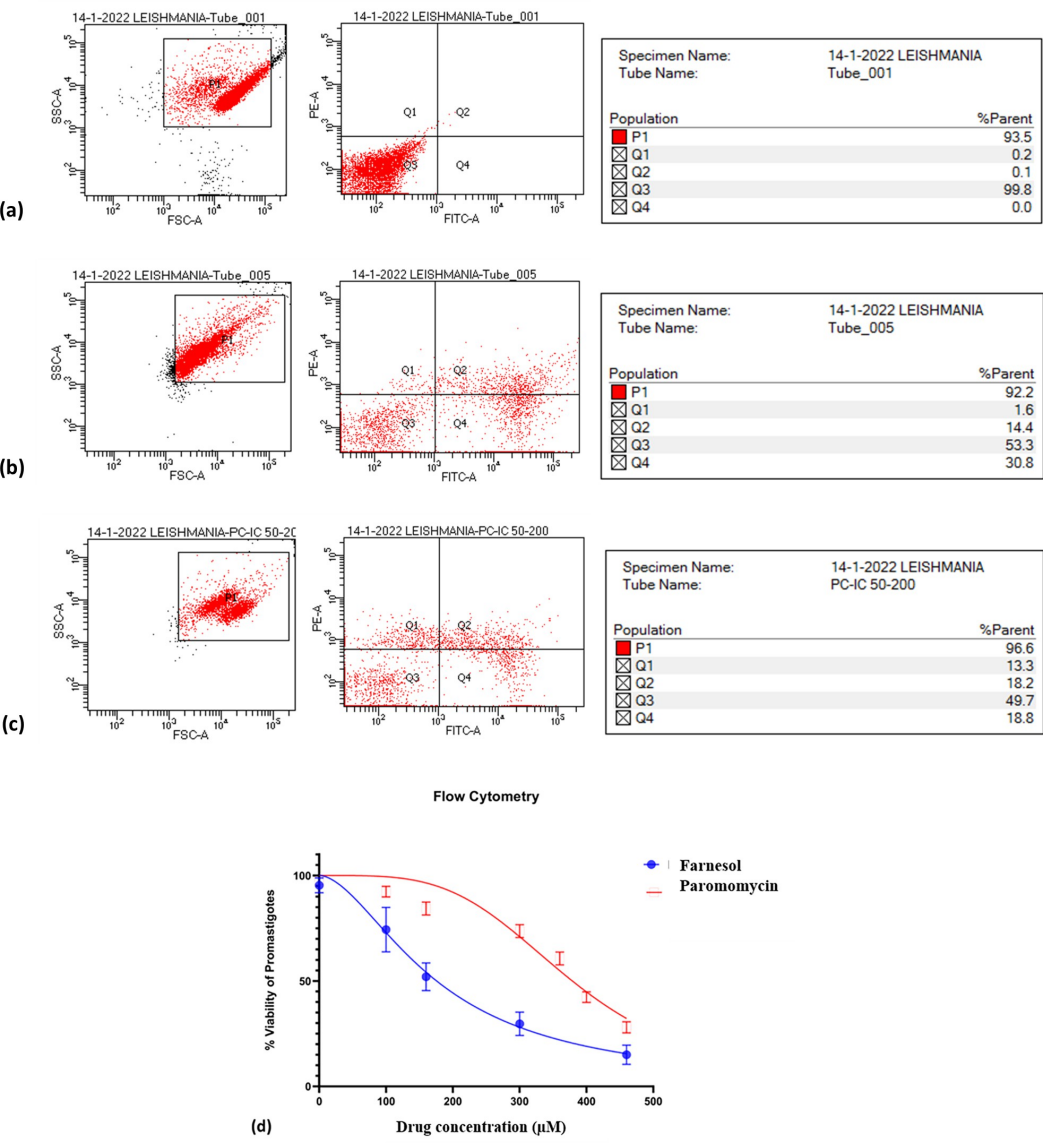
Flow cytometry analysis- A. control showing 99.8% live cells, B. 50% inhibition of *L*.*major* promastigotes on addition of Farnesol at 175.μM/ml against C. Paromomycin at 378.3μM D. Graph showing IC_50_ values of drugs against promastigotes of *L*.*major* by flow cytometry.

The purpose of this experiment was to determine the percentage of necrotic, apoptotic, and normal cells caused by the drugs activity (100–400 μM/ mL). After 72 hours of incubation, Farnesol was observed to decrease *L*.*major* promastigotes. At 175.7 ± 1.7 μM, normal, necrotic, and apoptotic promastigotes were estimated to account for 54%, 5.53%, and 40.43% of the total. The status of the control group was determined to be 99.2%, 0.021%, and 0.731%, respectively (Fig 3). Farnesol’s treatment caused apoptosis in promastigotes.

### 2.3 Cytotoxicity of farnesol to macrophages by MTT

**Fig 4:**
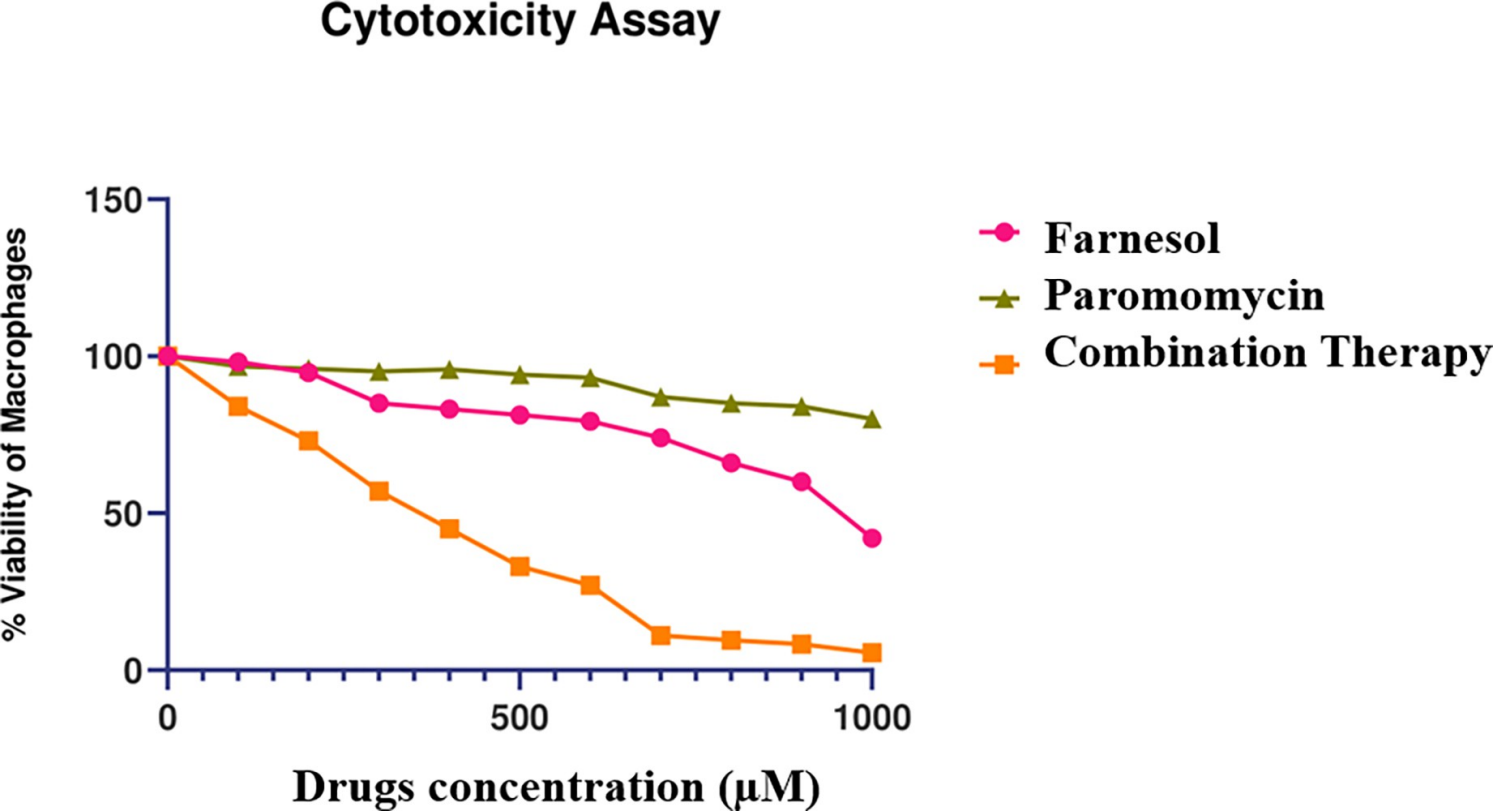
Farnesol cytotoxicity assay on THP 1 macrophages showing percentage viability of macrophages against different drug concentrations.

After 72 hours of incubation, macrophages subjected to various dosages of Farnesol showed 50% killing at 945 μM compared to Paromomycin at 362 μM and the S.I. of Farnesol (5.65) was relatively five times greater than the S.I. of Paromomycin (1.09) (Fig 4).

### 2.4 Amastigote assay

**Fig 5:**
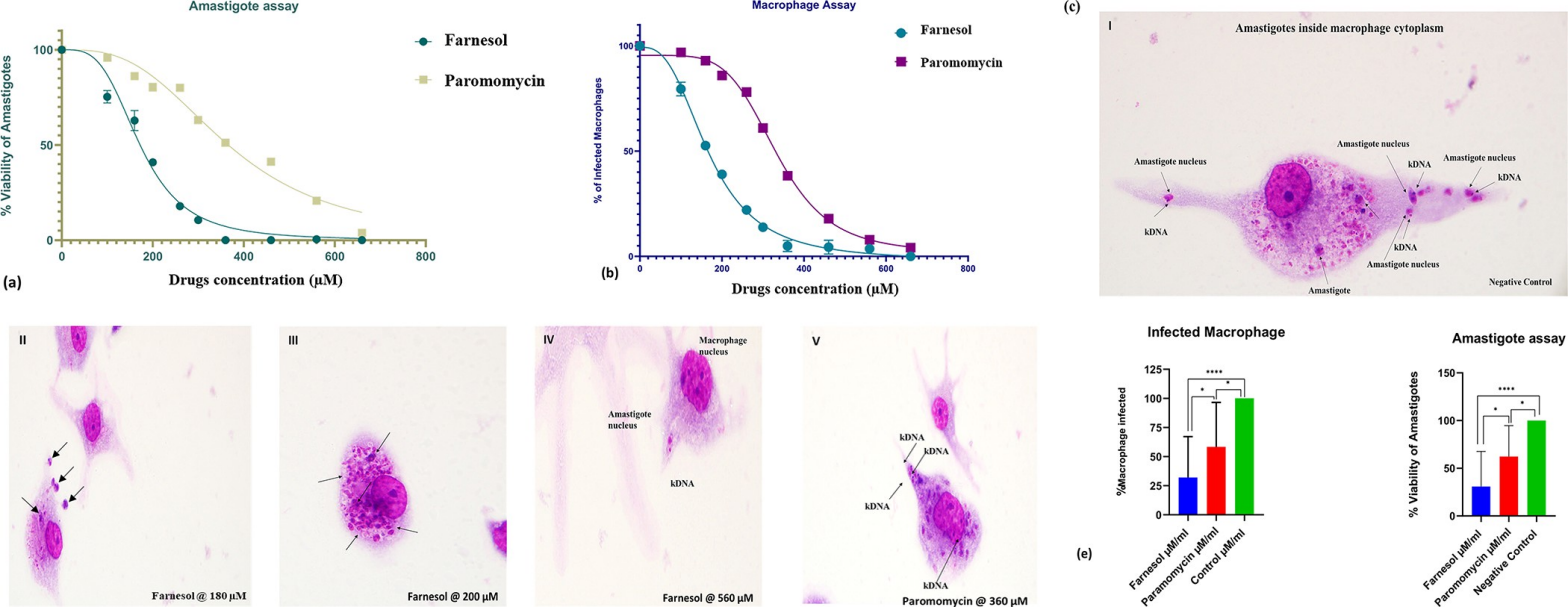
**a.** IC_50_ values by Amastigotes assay **b.** IC_50_ values by Macrophage assay **c.** Macrophages infected by *L*.*major* amastigotes after treatment at different concentrations of Farnesol, **d.** All statistical tests were two-tailed, with a significance level of p<0.05,* p<0.05,** p<0.01, *** p<0.001 & **** p<0.0001 was considered statistically significant.

Light Microscopy was employed to evaluate the rate of infected macrophages based on the number of infected cells in the negative and positive control slides (Fig 5C I). Fourty three percent (43%) of macrophages were infected by Paromomycin (100–560 μM), but infection rates in macrophages treated with Farnesol at 160 and 200 μM concentrations were 29% and 23%, respectively. In the positive control slides, however, 55% of the macrophages were infected. These findings demonstrate Farnesol’s anti-amastigote properties, with an IC_50_ value of 172.3 ± 2.2μM & IC_90_ at 328.80 ± 2.4μM & Paromomycin-IC_50_ at 366.0 ± 2.5μM & IC_90_ 780.78 ± 2.89μM (Table 2 and Fig 5).

**Table 2:** Amastigote assay–Table showing the mean no. of amastigotes per 100 macrophages and the percentage of Macrophages infected after addition of drugs at different concentrations for 72 hours.

Conc. of Farnesol (μM)	Amastigote rate (per 100 macrophages)	% of Macrophages infected
100	130±3.29	44±3.11
160	110±5.27	29±3.66
200	72±1.39	23±2.28
260	31.33±2.178	15±2.8
300	17.66±1.17	7.66±5.94
360	7±2.33	3±2.64
460	2.33±3.30	2.3±3.26
560	1±0.33	2±1.8
Paromomycin at 366μM	89.667±4.5	43.4±2
Positive Control	175±3.1	55.66±5.13

### 2.5 Combination therapy

**Fig 6:**
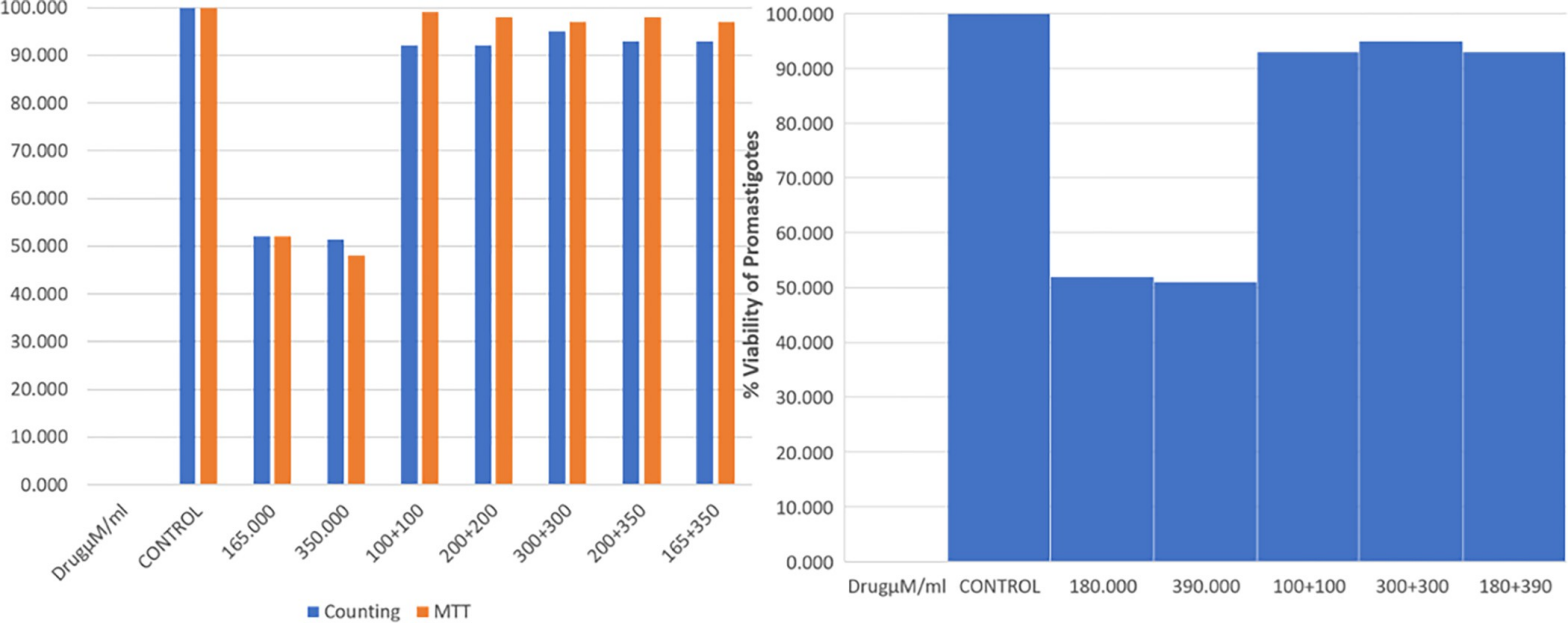
Combination therapy of Farnesol & Paromomycin on *L*.*major* promastigotes: The graph shows that Paromomycin and Farnesol capped each other’s killing activity, as they displayed normal killing effects when treated separately with Farnesol at 167μM and Paromomycin at 332μM but the combination of both drugs reduced their combined ability to fight off infection, for both promastigotes or amastigotes assays.

Both drugs had no effect on lesion healing. Paromomycin and Farnesol capped each other’s antiparasitic activity, as they displayed normal killing effects when treated separately with Farnesol at 167μM and Paromomycin at 332μM but the combination of both drugs reduced their combined ability to fight off infection, for both promastigotes or amastigotes assays (Fig 6). The combination therapy gave no significant healing response.

### 2.6 *In silico* drug-binding assay for tracing the inhibitory action of Farnesol

**Fig 7:**
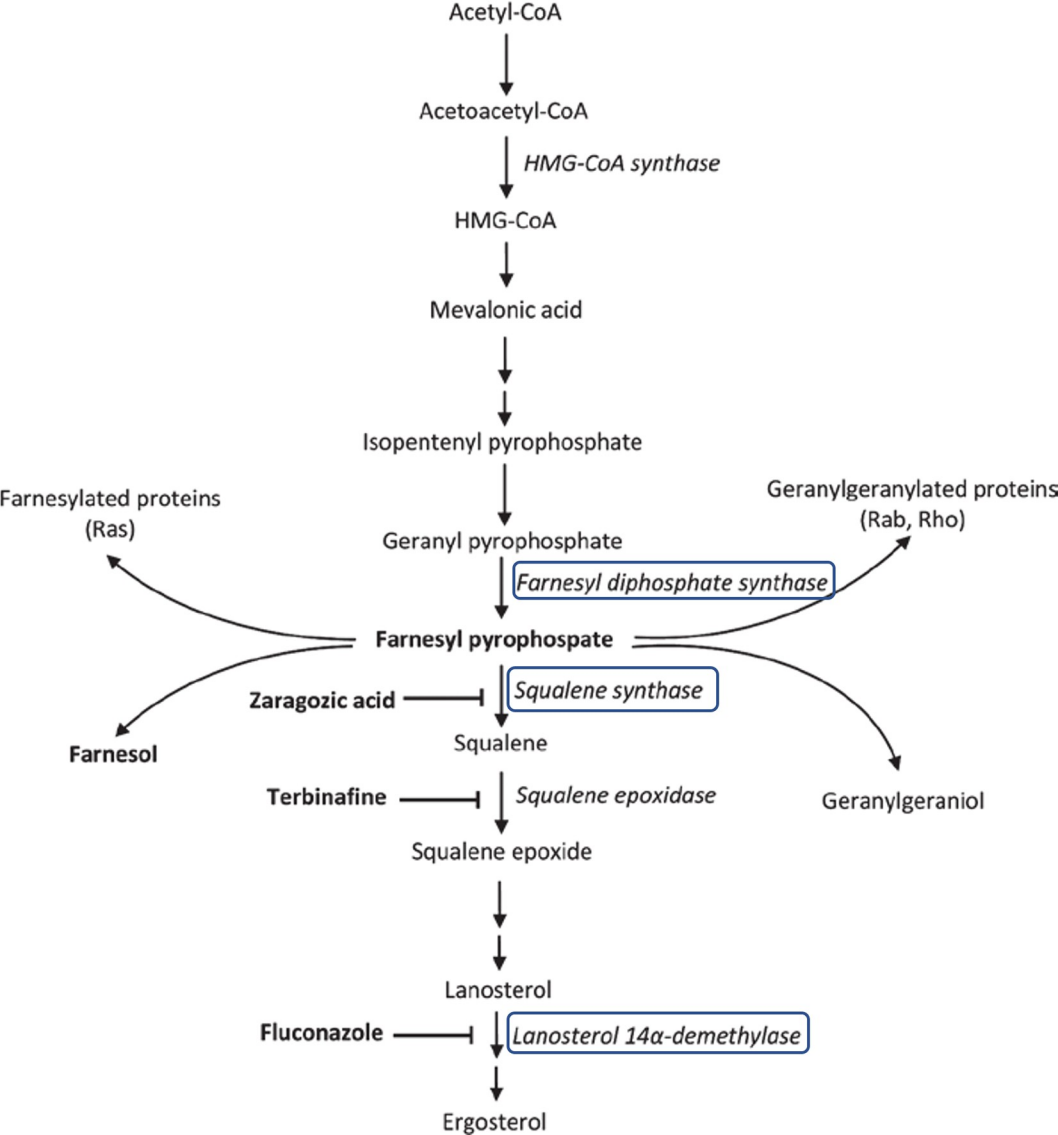
Ergosterol synthesis pathway and the 3 key inhibitors chosen for the study [26].

**Fig 8:**
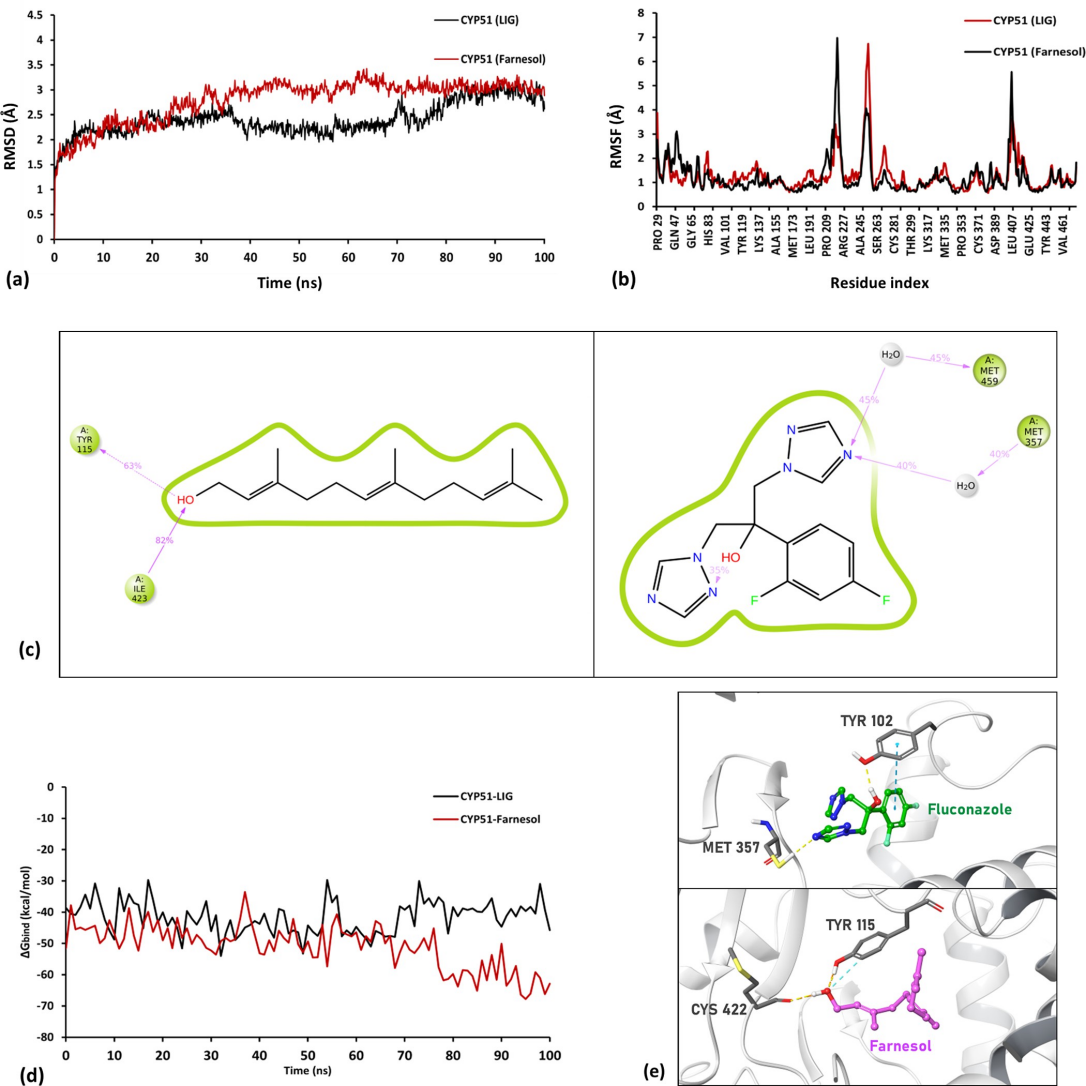
*In silico* binding profile; (a) Root mean square deviation (RMSD) of the 100ns molecular dynamics (MD) run of Farnesol-CYP51(red) and fluconazole (or LIG)-CYP51 (black) protein-ligand (P-L) complexes, (b) Root mean square fluctuation of respective CYP51-ligand complexes in 100ns MD run, (c) Protein-ligand interaction summary of 100ns MD run where I shows Farnesol and II shows fluconazole as ligand in centre, (d) ΔG_*bind*_ free energy change of respective CYP51-ligand complexes in 100ns MD run calculated using *thermal_mmgbsa*.*py* script provided by Schrodinger Inc., (e) Docked poses of fluconazole and Farnesol with CYP51 active site with dotted lines representing different P-L interactions (yellow: H-bond, teal: Aromatic interaction, orange: Hydrophobic contacts).

In previous studies, Farnesol accumulation has been linked to the inhibition of ergosterol synthesis in *Coccidiodes* spp., which ultimately resulted in its antifungal activity **[25]**. Based on available literature, we selected three crucial enzymes of the ergosterol synthesis present study: Farnesol pyrophosphate synthase (FPPS) [PDBid: 4K10], Squalene synthase [modbase model], and Lanosterol 14-demethylase (CYP51) [modbase model]to assess Farnesol’s binding in comparison to respective inhibitors/substrates of the mentioned enzymes (Fig 7) (Table 3).Our results from molecular docking suggests the affinity of Farnesol was more inclined towards CYP51 in comparison to the other two target enzymes. Moreover, Farnesol surpasses the intrinsic inhibitor, fluconazole in the binding scores. To further look into the efficacy of Farnesol in engaging the active site of CYP51, the MD simulations of the respective protein-ligand (P-L)-complexes were performed for 100ns under the conditions- NPT ensembles, TIP3P water model, at 310°C and 1Pa pressure- to further examine the effective binding of Farnesol with CYP51 over FPPS and Squalene synthase reported in docking and MM-GBSA scores. It was found that Farnesol displays contrastingly efficient interaction with the CYP51 receptor, i.e., stable binding with CYP51 in comparison to fluconazole, as seen in Fig 8. In the case of Farnesol, the root mean square deviation (RMSD) of the P-L complexes may be shown to be stabilizing. Though the molecular docking score of fluconazole was lower than Farnesol, tbinding energy of Farnesol was significantly lower than fluconazole, hence stronger complex formation.

**Table 3:** Docking score to assess Farnesol binding in comparison to respective inhibitors/substrates of the mentioned enzymes.

Enzymes	Docking score	ΔG_*bind*_ (kcal/mol)	Docking score	ΔG_*bind*_ (kcal/mol)
FPPS	Farnesol		Farnesyl pyrophosphate	
	-3.51	-32.6	-6.24	-44.3
Squalene synthase	Farnesol		Farnesyl thiopyrophosphate	
	-3.04	-38.52	-9.38	-38.31
Lanosterol 14-α demethylase	Farnesol		Fluconazole	
	-5.10	-49.75	-6.9	-41.02

Furthermore, it has greater interaction percentages than fluconazole due to its better H-bond formation with Tyr115 and Ile423 (Fig 8C). Also, the binding free energy change of MD trajectory (Fig 8D) exhibit similar results, it can be seen further declining for the last 30ns of simulation up to -69.8 kcal/mol in comparison to fluconazole which only lies around -46 kcal/mol (Fig 8) **[27]**.

## Discussion

*Leishmania* infections pose a significant challenge in infectious biology, necessitating the exploration of novel treatments and drugs. The distinct nature of CL and VL presents unique challenges in terms of drug distribution, with cutaneous cases requiring dermal distribution and visceral cases requiring extensive tissue penetration [28]. In recent years, there has been growing interest in plant-based compounds as potential therapeutics for leishmaniasis. Farnesol, a sesquiterpene derived from plants, has emerged as a compound with diverse biological and therapeutic applications (28). One notable advantage of Farnesol is its low toxicity compared to other chemically derived anti-leishmanial drugs such as Gentamycin and Miltefosine. This characteristic makes Farnesol an attractive candidate for further investigation. Farnesol, known as a self-secreted quorum-sensing molecule, has shown promise in various biological activities, including antiviral, anticancer, and anti-protozoan properties. Its potential role in inhibiting apoptosis further adds to its therapeutic relevance. Farnesol’s pharmacokinetic profile, characterized by a Log kp value of -3.81 cm/s and high lipophilicity (Log Po/w = 4.32), suggests its ability to penetrate membrane barriers and reach intracellular amastigotes [29].

Our study aimed to assess the anti-leishmanial activity of Farnesol using *in vitro* assays. The MTT assay revealed dose-dependent susceptibility of *L*.*major* promastigotes and amastigotes to Farnesol. Remarkably, the IC_50_ and IC_90_ values of Farnesol were approximately half of those observed for the FDA-approved drug Paromomycin, indicating its potent anti-leishmanial efficacy (Table 4).

**Table 4:** Comparisons of both drugs for different assays performed to evaluate the IC_50_ and IC_90_ values against *L*.*major* parasites.(ND- Not defined).

Drug assays		Farnesol (test drug)		Paromomycin (control drug)	
		IC-50μM	IC-90 μM	IC-50 μM	IC-90 μM
**Promastigote Assay**	**MTT**	167.6±4.5	273.10±2.44	332.0±5.1	510.79±2.71
	**Counting**	170.5±4.2	287.69±2.46	353.2±3.7	639.62±2.81
**Amastigote assay**	**%Macrophage infected**	169.1±2.8	370.63±2.57	334.3±5.1	514.33±2.71
**Flow Cytometry**	**Amastigote /100 macrophages**	172.3±2.2 175.7±1.7	328.80±2.4 ND	366.0±2.5 378.8±3.8	780.78±2.89 ND

Additionally, Farnesol-induced apoptosis was observed in *L*.*major* promastigotes, suggesting its involvement in inhibiting parasite growth. While the precise mechanism of Farnesol’s anti-leishmanial activity remains unknown, existing research on its antifungal effect points to its influence on sterol biosynthesis [27]. To investigate this further, we conducted *in silico* protein-ligand interaction studies, which revealed potential interactions between Farnesol and Lanosterol 14-demethylase, a key enzyme in the ergosterol production pathway.

Our results from molecular docking indicated that Farnesol exhibited a greater affinity for CYP51 compared to the other two target enzymes. Notably, Farnesol showed superior binding scores compared to the intrinsic inhibitor fluconazole. To further evaluate the effectiveness of Farnesol in engaging the active site of CYP51, we performed MD simulations of the protein-ligand complexes. These simulations provided valuable insights into the stability and interaction dynamics of Farnesol with CYP51 over an extended period of time. Although the molecular docking score of fluconazole was lower than that of Farnesol, the binding energy of Farnesol was significantly lower than that of fluconazole. This indicates that Farnesol forms a strong and stable complex with CYP51, potentially contributing to its potent anti-leishmanial activity. It is important to note that the molecular docking scores are based on shape-complementarity, while the MM-GBSA free energy calculations rely on the ΔG_*bind*_ free energy change of complex formation the MM-GBSA approach is considered more reliable in assessing binding affinities [30].

The MD simulations of the docked complexes revealed that Farnesol displayed efficient and stable binding with CYP51, as evidenced by the equilibrating RMSD of the protein-ligand complexes. Further trajectory analysis revealed a consistent decrease in the ΔG_*bind*_ free energy change for the CYP51-Farnesol complex, providing additional support for the efficacy of Farnesol as an effective inhibitor of the ergosterol synthesis pathway (Fig 8C).

These results in components do complement the *in vitro* findings demonstrating the potent anti-leishmanial activity of Farnesol against *L*. *major* promastigotes and amastigotes. Nonetheless, further studies are needed to validate these findings and investigate the efficacy of Farnesol *in vivo*. Additionally, optimizing the formulation and conducting pharmacokinetic studies will be crucial steps towards the development of Farnesol as a potential therapeutic agent for CL. Overall, our study highlights the multifaceted potential of Farnesol as a promising alternative for the treatment of leishmaniasis, and further research in this area is warranted to unlock its full therapeutic potential.

To the best of our knowledge, this study represents the first exploration of Farnesol’s effects on CL. The significant anti-leishmanial potential of Farnesol, as evidenced by its potent efficacy against *L*.*major* parasites and its favourable pharmacokinetic profile, warrants further investigation and development. Future studies should focus on elucidating the precise molecular mechanisms of Farnesol’s action, conducting *in vivo* experiments to validate its therapeutic efficacy, and optimizing its formulation for clinical applications.

## Conclusion

In conclusion, the present study reveals the potential of Farnesol as a promising therapeutic agent for leishmaniasis. Farnesol demonstrated potent anti-leishmanial activity against *L*.*major* parasites, with superior efficacy compared to the standard drug Paromomycin. *In vitro* assays confirmed dose-dependent susceptibility and Farnesol-induced apoptosis in the parasites. *In silico* studies suggested Farnesol’s interaction with Lanosterol 14-demethylase, a key enzyme in the ergosterol production pathway. Molecular docking and MD simulations supported the stable binding of Farnesol with CYP51, further emphasizing its potential as an effective inhibitor of ergosterol synthesis. Further the high Log Po/w score suggests its strong transmembrane mobility and ability to interact with intracellular amastigotes. Our findings highlight the multifaceted therapeutic potential of Farnesol and call for further research to validate its efficacy *in vivo* and optimize its formulation for clinical applications.
